# Very Rare Case of Hyaline Fibromatosis Syndrome Successfully Treated with Surgical Excision and Review of Literature

**DOI:** 10.1055/a-2665-2209

**Published:** 2025-11-20

**Authors:** Yong Uk Jung, Byung-jun Kim, Eun-Hee Kim

**Affiliations:** 1 Department of Plastic and Reconstructive Surgery, Seoul National University Hospital, Jongno-gu, Seoul, South Korea; 2 Department of Anesthesiology and Pain Medicine, Seoul National University Hospital, Jongno-gu, Seoul, South Korea

**Keywords:** hyaline fibromatosis syndrome, skin graft, reconstruction, review of literature

## Abstract

Hyaline fibromatosis syndrome (HFS) is a rare autosomal recessive disorder caused by *ANTXR2* gene mutations, resulting in abnormal deposition of hyaline material in connective tissues. Fewer than 100 cases have been documented worldwide. Patients often present with painful joint contractures, gingival hyperplasia, cutaneous nodules, and restricted mobility. Here, we report a 7-year-old boy with HFS who underwent surgical management for near-total obstruction of the external ear canal and multiple ulcerative lesions. A multidisciplinary approach facilitated successful mass excision and reconstruction with a split-thickness skin graft. Postoperative evaluations showed stable wound healing at 6 months, allowing consideration of further surgeries. This case highlights the importance of comprehensive genetic assessment, careful preoperative planning, and individualized surgical intervention, as well as the critical role of nutritional support to optimize wound healing and clinical outcomes in HFS.

## Introduction

Hyaline fibromatosis syndrome (HFS) represents an autosomal recessive condition marked by excessive deposition of hyaline material in connective tissues across multiple body systems. This rare disorder, caused by mutations in the *ANTXR2* gene, presents in two forms: The severe infantile systemic hyalinosis (ISH) and the milder juvenile hyaline fibromatosis (JHF). 1 With only around 100 documented cases worldwide, 2 HFS is characterized by significant morbidity, and its management requires a comprehensive understanding of the underlying pathology and potential complications. The unification of ISH and JHF into a single entity, proposed in 2009 by Nofal et al., 3 highlights the similar genetic underpinnings of these forms, though they differ in presentation and severity.

Historically, HFS was first identified by Murray in 1873 under the term “molluscum fibrosum,” 4 and it has since posed considerable challenges in clinical management. There is a dearth of literature on effective surgical management and postoperative outcomes, particularly concerning wound healing and the impacts of tissue structure on graft uptake. This case report contributes to the existing literature by detailing the surgical and postoperative course of HFS in a pediatric patient.

## Case

The patient and parents provided written informed consent for the use of the patient's medical records and photographs in this case report.

**Fig. 1:**
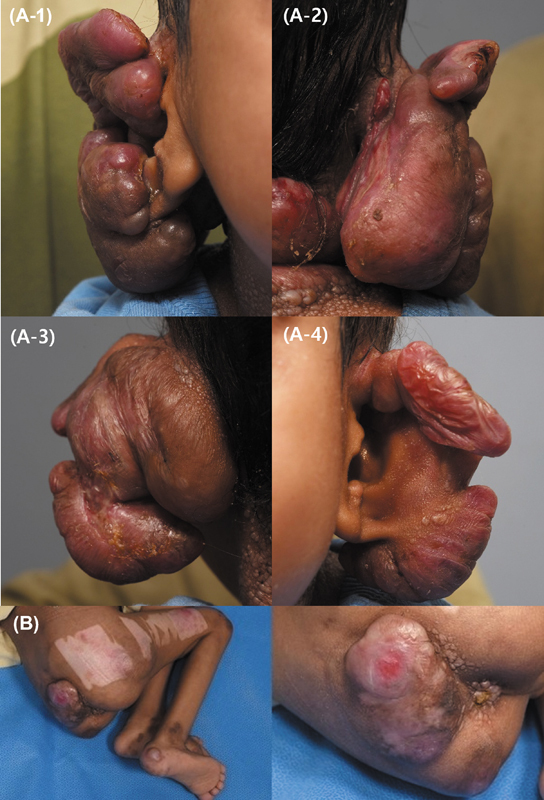
Preoperative photograph of the patient's ear. **(A-1, A-2)** Anterior and posterior views of the right ear. **(A-3, A-4)** Anterior and posterior views of the left ear **(A)** . Preoperative image of the patient's sacrum, captured following two prior surgical procedures **(B)** .

A 7-year-old male with genetically confirmed HFS was referred to plastic surgery for management of ulcerative skin lesions and an obstructive posterior ear mass. The mass caused partial blockage of the external auditory meatus, potentially leading to complications in hearing and further treatment. Additional ulcerative lesions were noted on the sacrum and neck, likely due to pressure-related issues from limited mobility ( Fig. 1 ). This case represents a relatively typical clinical manifestation of HFS.

Preoperative considerations focused on airway management and patient positioning due to the complex anatomical features associated with HFS. Joint contractures, temporomandibular stiffness, and hyaline deposition in the oral and nasal cavities posed significant challenges for intubation. A pediatric otolaryngologist conducted a detailed airway assessment, and a computed tomography scan was obtained to evaluate airway patency and identify potential obstructions. Given the limited mouth opening and gingival hyperplasia, nasotracheal intubation under fiberoptic guidance was selected, with emergency tracheostomy prepared as a contingency.

Patient positioning required meticulous planning. Cervical spine contractures necessitated specialized support to ensure stability and comfort during the procedure. The left lateral decubitus position provided optimal surgical access to the affected ear while minimizing strain on contracted joints. Additional padding was applied to prevent pressure-related complications, particularly over previously affected areas such as the sacrum.

**Fig. 2:**
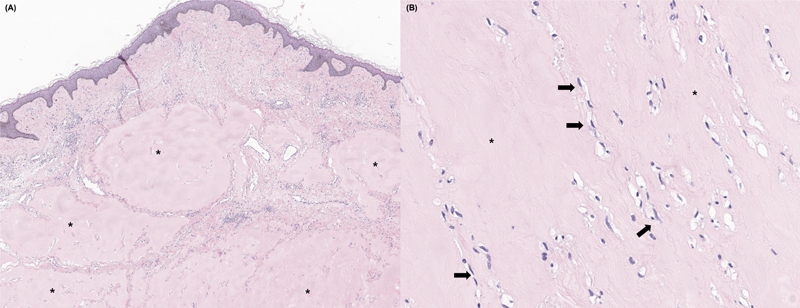
Histopathological examination of the excised lesion. **(A)** A low-power photomicrograph reveals abundant deposition of eosinophilic hyaline material within the dermis, indicated by asterisks. This homogenous, glassy material is characteristic of hyaline fibromatosis syndrome and stains prominently with hematoxylin and eosin. **(B)** A high-power image demonstrates numerous mononuclear, spindle-shaped fibroblasts (arrows) embedded within the eosinophilic hyaline matrix (asterisks). These findings are consistent with the diagnostic histological features of juvenile hyaline fibromatosis (hematoxylin–eosin stain).

**Fig. 3:**
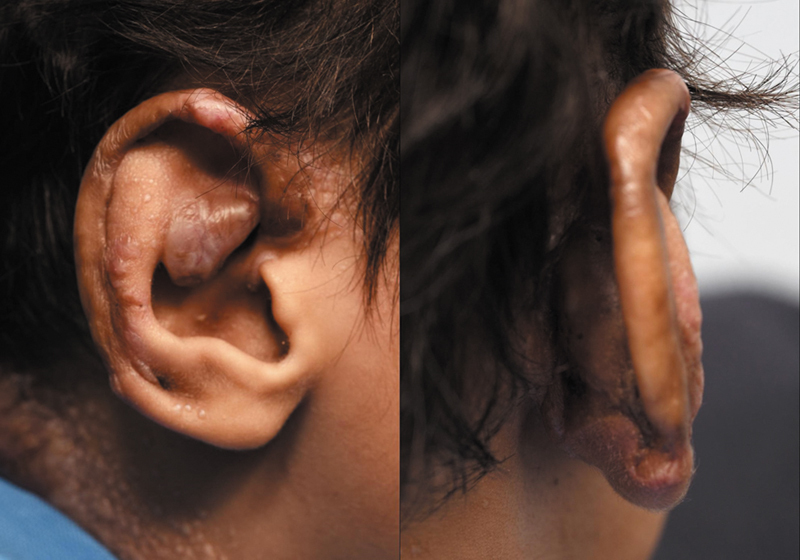
Photograph of the patient's right ear taken 6 months postoperatively, demonstrating complete healing with no complications observed.

Surgical intervention included excision of the auricular mass followed by reconstruction using a split-thickness skin graft harvested from the left lateral thigh. A tie-over dressing was applied to enhance graft adherence. Histopathological analysis confirmed a diagnosis of JHF ( Fig. 2 ). The patient exhibited satisfactory wound healing over 6 months ( Fig. 3 ), permitting subsequent staged procedures. These included excision and reconstruction of lesions on the heel and sacral regions utilizing local flaps, with a primary focus on tension-free closure to facilitate optimal healing.

Following initial procedures, additional surgeries were performed to excise fibrous masses on the buttock, neck, and heel. At 20 months postoperatively, a recurrent mass with overlying inflammation appeared on the right auricle, necessitating another excision and skin grafting, which was completed without complications. Recurrence was noted within 2 months following the initial surgery. Although gross total excision was achieved during the first operation, a microscopic residual lesion is presumed to have led to recurrence. During the second procedure, dissection from the perichondrium was performed without difficulty, and no significant adhesions or unusual intraoperative findings were observed. Intraoperative bleeding was minimal and within the expected range for this type of surgery.

At the age of 2, a gastrostomy tube was placed due to feeding difficulties; however, site bleeding prompted further evaluation with esophagogastroduodenoscopy and consultation with a nutrition specialist. The patient continues to require multidisciplinary management, including physiotherapy from the Department of Rehabilitation Medicine, orthopedic follow-up for knee contractures, and otolaryngologic surveillance of airway patency.

## Discussion

While some authors differentiate ISH from JHF, both present overlapping clinical features like skin lesions, subcutaneous masses, gingival hyperplasia, joint stiffness, and osteopenia. 5 ISH, however, often involves higher mortality due to complications like severe diarrhea and recurrent bacteremia. 6 Despite clinical differences, both syndromes share the same *ANTXR2* gene mutation, supporting the unified term “HFS.” 1 Based on symptom presentation, our patient's HFS aligns with grade 2 (moderate severity). 3

Neurofibromatosis, gingival hyperplasia, nodular amyloidosis, Winchester syndrome, and lipoid proteinosis, along with inborn metabolic disorders such as Farber's disease, mucopolysaccharidoses, and I-cell disease, could be considered in the differential diagnosis of HFS. 7 Although these conditions share clinical features with HFS, the underlying genetic mutations responsible for each condition are distinct. Therefore, a genetic analysis is crucial for accurate differential diagnosis and appropriate management.

Standard treatments for joint contractures, such as intralesional or systemic steroid therapy and oral D-penicillamine, 8 often yield limited results. Steroid therapy may cause symptom recurrence, and in our case, inflammatory wound conditions contraindicate its use. A review of several articles on HFS treatment, including surgical approaches ( Table 1 ), 8 9 10 11 12 13 14 15 led us to conclude that, despite concerns about surgical intervention potentially stimulating further hyaline deposition, excision of painful or infected lesions can provide substantial symptomatic relief. This finding is supported by previous studies 9 11 13 and was observed in our patient as well. Surgical excision should be viewed as palliative, not curative, due to frequent recurrence. 10 Unlike keloid formation, where recurrence often occurs shortly after surgery, HFS typically exhibits a remission period postexcision, allowing surgeons to perform excisions with the goal of palliation.

**Table 1:** Summary of reviewed literature: Most reports are case studies. Successful mass excision is common, though recurrence remains frequent 8 9 10 11 12 13 14 15

Study	Year	Ethnicity	Number of patients/sample size	Treatment modality	Location of lesion	Follow-up period	Outcomes	Comments
Woyke et al. 9	1984	Not specified	2 cases	Surgical excision	Scalp, shoulders, back, and extremities. gingivae, face, hand, and fingers.	19 years	Successful removal of nearly all masses; recurrence frequently reported	–
El-Maaytah et al. 8	2010	Middle Eastern	1 case	Surgical excision+ Oral D-penicillamine+ Physiotherapy	Scalp, gingivae, face, and left arm	15 years	Successful removal of masses, though recurrence frequently reported. Joint contracture improved.	Duration of D-penicillamine treatment was not specified.
Krishnamurthy et al. 10	2011	Not specified	1 case	Surgical excision	Scalp, back, face, chest, and abdomen	7 years	Successful removal of masses; recurrence frequently reported	–
Marques et al. 11	2016	Not specified	1 case	Surgical excision	Shoulder, back, face, arm, and finger	20 years	Successful removal of nearly all masses; recurrence frequently reported	Only 1 patient out of 2 had treatment and follow-up history.
Baltacioglu et al. 12	2017	Not specified	1 case	Surgical excision+ Systemic steroid	Gingivae, face, and perianal region	10 years	Successful removal of nearly all masses; recurrence frequently reported	Steroid effect not mentioned
Braizat et al. 13	2020	African	1 case	Surgical excision+ Systemic and Intralesional steroid+ Physiotherapy	Gingivae, face, hand, back, and big toe	5 years	Successful removal of nearly all masses. Recurrence reported. Systemic and intralesional steroids were effective, but physiotherapy was not	Recurrence was confined to hand lesions.
Song et al. 14	2021	Not specified	5 cases	Surgical excision	Scalp, face, hand, knee, back, shoulder, elbow, arm, thigh, and foot	1–10 years	Successful removal of nearly all masses. Recurrence reported	One patient in the case series lacked recurrence details.
Chaisrisawadisuk et al. 15	2024	Not specified	1 case	Surgical excision	Scalp, gingivae	4 month	Successful removal of mass; no recurrence	–

Given the compromised skin condition, primary closure was not viable; split-thickness skin grafting was used. While no specific studies address graft uptake in HFS, our patient's graft integrated successfully, indicating hyaline deposition may not hinder short-term outcomes. Physiotherapy did not benefit our patient, as joint splints caused discomfort and no noticeable improvements were observed, consistent with previous reports on HFS. In contrast, nutritional support played a critical role in the patient's recovery, 16 particularly given the gastrointestinal complications common in HFS, such as villous atrophy, intestinal edema, and lymphangiectasia, which can lead to chronic diarrhea. 3 These conditions increase the risk of malnutrition, potentially hindering wound healing and recovery. Proper nutrition facilitated wound healing, particularly after surgical interventions, and proved essential for maintaining overall stability in the patient's health.

Airway management was particularly challenging due to temporomandibular and cervical joint contractures, along with hyaline deposits in the oral, nasal, and tracheal areas. 17 18 19 Thorough preoperative assessment by pediatric otolaryngology and anesthesiology, including CT imaging for airway patency, was essential. 17 18 Although guidelines recommend conscious intubation for difficult airways, 20 this approach is often impractical in young children. Limited mouth opening and gingival hyperplasia precluded standard airway device use, making nasal fiberoptic intubation the preferred option. Despite visualization challenges from hypertrophied soft tissues, nasotracheal intubation was successful. An emergency tracheostomy setup was prepared, and extubation was performed with caution, ready for emergent reintubation if needed.

Our case report presents several limitations. It lacks a pathological analysis of the postoperative wounds related to the skin graft, which complicates the assessment of whether the graft uptake process corresponds with the typical postoperative course observed in skin grafts. Additionally, we opted not to utilize local or systemic steroid therapy due to the patient's wound condition, leaving the potential effects of steroid therapy on the healing process unexplored within the context of this case. Lastly, as this is a case report, it underscores the necessity of accumulating clinical experience to foster a more comprehensive understanding of HFS.

### Conclusion

Managing HFS involves addressing both the unique pathophysiological challenges and the complex postoperative needs associated with this syndrome. Genetic testing is imperative for accurate diagnosis, and multidisciplinary care is essential for optimizing outcomes. This case report highlights the efficacy of split-thickness skin grafting and tension-free reconstruction in HFS patients and reinforces the importance of comprehensive nutritional support and preoperative planning. Surgical intervention can provide symptomatic relief, but recurrence is common, necessitating clear patient communication regarding long-term management.

## References

[1] Mantri M D Pradeep M M Kalpesh P O Pranavsinh R J Hyaline fibromatosis syndrome: A rare inherited disorder Indian J Dermatol 2016 61 05 580 10.4103/0019-5154.190129 PMC5029258 27688461

[2] Xia L Hu Y Zhang C Wu D Chen Y Juvenile hyaline fibromatosis: a rare oral disease case report and literature review Transl Pediatr 2021 10 11 3124 3129 34976780 10.21037/tp-21-169 PMC8649598

[3] Nofal A Sanad M Assaf M Juvenile hyaline fibromatosis and infantile systemic hyalinosis: a unifying term and a proposed grading system J Am Acad Dermatol 2009 61 04 695 700 19344977 10.1016/j.jaad.2009.01.039

[4] Murray J On three peculiar cases of molluscum fibrosum in children in which one or more of the following conditions were observed: Hypertrophy of the gums, enlargement of the ends of the fingers and toes, numerous connective-tissue tumours on the scalp, &c Med Chir Trans 1873 56 235 254.1 PMC1988914 20896407

[5] Thomas J E Moossavi M Mehregan D R McFalda W L Mahon M J Juvenile hyaline fibromatosis: A case report and review of the literature Int J Dermatol 2004 43 11 785 789 15533058 10.1111/j.1365-4632.2004.02239.x

[6] Stucki U Spycher M A Eich G Infantile systemic hyalinosis in siblings: Clinical report, biochemical and ultrastructural findings, and review of the literature Am J Med Genet 2001 100 02 122 129 11298373 10.1002/1096-8628(20010422)100:2<122::aid-ajmg1236>3.0.co;2-0

[7] Dhingra M Amladi S Savant S Nayak C Juvenile hyaline fibromatosis and infantile systemic hyalinosis: Divergent expressions of the same genetic defect? Indian J Dermatol Venereol Leprol 2008 74 04 371 374 18797061 10.4103/0378-6323.42913

[8] El-Maaytah M Jerjes W Shah P Upile T Murphy C Ayliffe P Gingival hyperplasia associated with juvenile hyaline fibromatosis: A case report and review of the literature J Oral Maxillofac Surg 2010 68 10 2604 2608 20863945 10.1016/j.joms.2009.09.060

[9] Woyke S Domagala W Markiewicz C A 19-year follow-up of multiple juvenile hyaline fibromatosis J Pediatr Surg 1984 19 03 302 304 6747795 10.1016/s0022-3468(84)80192-x

[10] Krishnamurthy J Dalal B S Gubanna M V Juvenile hyaline fibromatosis Indian J Dermatol 2011 56 06 731 733 22345782 10.4103/0019-5154.91840 PMC3276908

[11] Marques S A Stolf H O Polizel J O Munhoz T Brandao M C Marques M E Hyaline fibromatosis syndrome: cutaneous manifestations An Bras Dermatol 2016 91 02 226 229 27192526 10.1590/abd1806-4841.20163799 PMC4861574

[12] Baltacioglu E Guzeldemir E Sukuroglu E Juvenile hyaline fibromatosis: A 10-year follow-up Indian J Dermatol 2017 62 02 210 212 28400645 10.4103/ijd.IJD_166_16 PMC5363149

[13] Braizat O Badran S Hammouda A Juvenile hyaline fibromatosis: Literature review and a case treated with surgical excision and corticosteroid Cureus 2020 12 10 e10823 33173631 10.7759/cureus.10823 PMC7645300

[14] Song L Yang J Liu J Wang J Juvenile hyaline fibromatosis: A clinicopathological study of five cases Ann Diagn Pathol 2021 55 151835 34624626 10.1016/j.anndiagpath.2021.151835

[15] Chaisrisawadisuk S Rattana-Arpa S Vathanophas V Sathienkijkanchai A Hyaline fibromatosis syndrome: early outcomes following major craniofacial mass excision J Craniofac Surg 2024 35 05 e492 e495 38847516 10.1097/SCS.0000000000010401

[16] Grada A Phillips T J Nutrition and cutaneous wound healing Clin Dermatol 2022 40 02 103 113 34844794 10.1016/j.clindermatol.2021.10.002

[17] Norman B Soni N Madden N Anaesthesia and juvenile hyaline fibromatosis Br J Anaesth 1996 76 01 163 166 8672362 10.1093/bja/76.1.163

[18] Yasuda A Miyazawa N Inoue E Imai T Shionoya Y Nakamura K Anesthetic management of a juvenile hyaline fibromatosis patient with trismus and cervical movement limitation Anesth Prog 2021 68 02 117 118 34185859 10.2344/anpr-68-01-04 PMC8258748

[19] Segal S Khanna A K Anesthetic management of a patient with juvenile hyaline fibromatosis: A case report written with the assistance of the large language model ChatGPT Cureus 2023 15 03 e35946 37038572 10.7759/cureus.35946 PMC10082625

[20] Apfelbaum J L Hagberg C A Connis R T 2022 American Society of Anesthesiologists Practice Guidelines for Management of the Difficult Airway Anesthesiology 2022 136 01 31 81 34762729 10.1097/ALN.0000000000004002

